# Cyberinfrastructure and resources to enable an integrative approach to studying forest trees

**DOI:** 10.1111/eva.12860

**Published:** 2019-11-03

**Authors:** Jill L. Wegrzyn, Taylor Falk, Emily Grau, Sean Buehler, Risharde Ramnath, Nic Herndon

**Affiliations:** ^1^ Department of Ecology and Evolutionary Biology University of Connecticut Storrs Connecticut

**Keywords:** cyberinfrastructure, FAIR, phenomics, plant ontologies, population genetics, tree databases, tree genomics, workflows

## Abstract

Sequencing technologies and bioinformatic approaches are now available to resolve the challenges associated with complex and heterozygous genomes. Increased access to less expensive and more effective instrumentation will contribute to a wealth of high‐quality plant genomes in the next few years. In the meantime, more than 370 tree species are associated with public projects in primary repositories that are interrogating expression profiles, identifying variants, or analyzing targeted capture without a high‐quality reference genome. Genomic data from these projects generates sequences that represent intermediate assemblies for transcriptomes and genomes. These data contribute to forest tree biology, but the associated sequence remains trapped in supplemental files that are poorly integrated in plant community databases and comparative genomic platforms. Successful implementation of life science cyberinfrastructure is improving data standards, ontologies, analytic workflows, and integrated database platforms for both model and non‐model plant species. Unique to forest trees with large populations that are long‐lived, outcrossing, and genetically diverse, the phenotypic and environmental metrics associated with georeferenced populations are just as important as the genomic data sampled for each individual. To address questions related to forest health and productivity, cyberinfrastructure must keep pace with the magnitude of genomic and phenomic sampling of larger populations. This review examines the current landscape of cyberinfrastructure, with an emphasis on best practices and resources to align community data with the Findable, Accessible, Interoperable, and Reusable (FAIR) guidelines.

## 1 INTRODUCTION

High‐throughput technologies are enabling rapid data generation in the fields of genomics, proteomics, and phenomics. These technological achievements are coupled with substantial reductions in cost as well as an increase in instrument accessibility. Genomics, in particular, exceeded predictions, and the cost of generating data is now less than the cost to store it (Stephens et al., 2015). High‐throughput genomic technologies encompass a myriad of short‐ and long‐read approaches that are competing to improve on read length, error rate, and cost per base. They enable biologists to increase the scale of their investigations, leading to larger populations, deeper coverage, more time points, and more replicates (Porter & Hajibabaei, 2018). This scaling is evident across all organismal systems and studies, encompassing metabarcoding, metagenomics, targeted capture, reduced representation sequencing, transcriptomics, epigenomics, and whole‐genome sequencing.

While we continue to celebrate decreasing sequencing costs, the associated expense of storing and analyzing large datasets is often outside the immediate calculus. The tremendous knowledge imparted by a reference genome is coupled with deep sequence data and information‐rich files associated with assembling and aligning billions of bases. The Sequence Read Archive (SRA), hosted by the National Center for Biotechnology (NCBI), provides a live accounting of the raw sequence data submitted. SRA currently reports over 26 petabases of sequence and represents only a fraction of the data generated worldwide (February 2019; Sayers et al., 2019). The process of generating a reference often requires thousands of hours of compute time on a High‐Performance Computing (HPC) cluster to bring those sequence reads from assembly to annotation, as documented for the first pine mega‐genome (Wegrzyn et al., 2014; Zimin et al., 2014). These intensive single project requirements produce significant institutional challenges related to data storage, transfer, and analysis. Computational research in the field of big data, which includes genomics, is focused on more efficient compression algorithms, binary file formats, and improved data transfer protocols to meet current demands (Muir et al., 2016).

**Figure 1:**
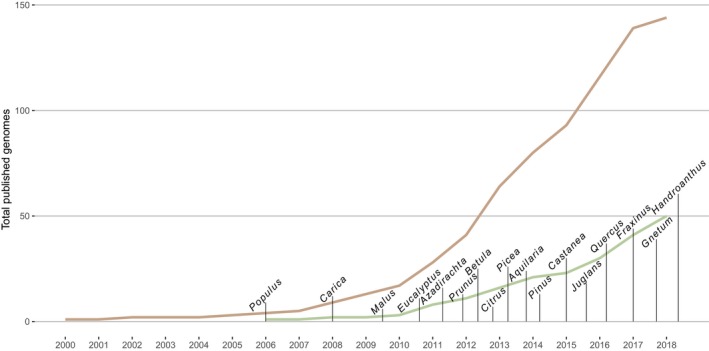
Growth in number of published reference plant genomes in comparison with those of tree species sequenced since 2002. By 2018, there were 148 plant reference genomes (shown in brown) with only 52 tree species (green). The first forest tree species was sequenced in 2006 (*Populus trichocarpa*). The highlighted genus names denote the year the first reference was generated for a species in that genus

**Figure 2:**
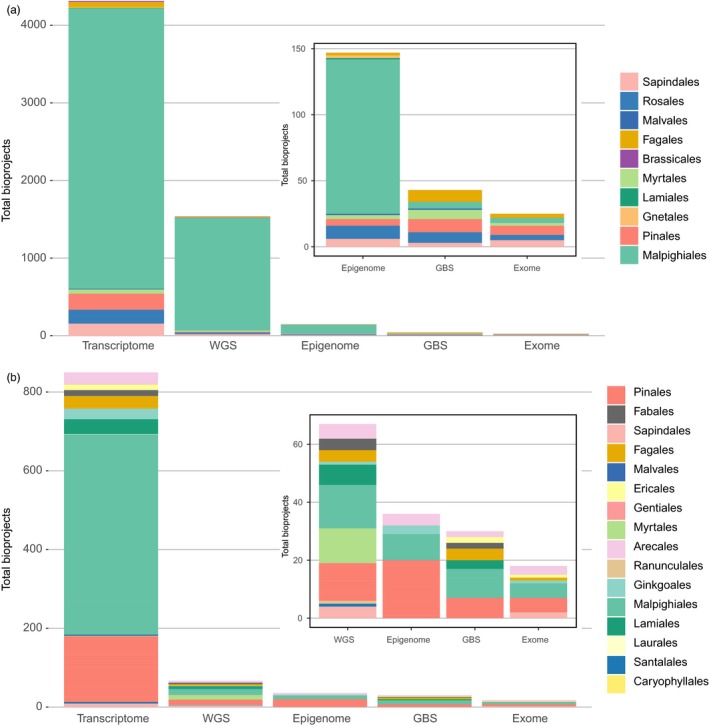
(a) NCBI project data depicted for 52 species (10 orders) associated with 6,116 BioProject studies. BioProject data were organized into whole‐genome shotgun (whole genome or resequencing), Transcriptome (RNA‐Seq, sRNA), Epigenome (bisulfite), GBS (genotyping‐by‐sequencing, RAD‐Seq, ddRAD‐Seq, RAPTURE, and similar), and exome (targeted capture). (b) NCBI BioProject data depicted for 972 projects representing 373 unique tree species across 16 orders. BioProject data were organized into whole‐genome shotgun (whole genome or resequencing), Transcriptome (RNA‐Seq, sRNA), Epigenome (bisulfite), GBS (genotyping‐by‐sequencing, RAD‐Seq, ddRAD‐Seq, RAPTURE, and similar), and exome (targeted capture)

Despite the increases in sequence‐based resources, fewer than 4,500 eukaryotic genomes are available in the NCBI Genome database. When examining the resources for vascular plants, just under 200 unique genomes are complete and 52 represent tree species (Figure 1). While full genomes are increasingly available, a significant amount of sequence data for forest trees remains associated with experiments that are not designed around a reference genome (Figure 2b). In contrast to the 52 species associated with over 6,100 NCBI BioProject studies, over 970 sequencing experiments represent 373 trees without a reference genome. The vast majority of this data is derived from genome sampling (i.e., GBS, RAD‐Seq) or transcriptomic approaches (Figure 2a). This leaves most forest tree species categorized as non‐model. The ability to achieve high‐quality reference genomes in forest trees is hindered by characteristics shared by other plant groups, including high heterozygosity, ploidy, gene duplications, and repetitive sequences (Hirsch & Robin Buell, 2013).

Recent advancements in optical mapping approaches, chromosome conformation capture, and long‐read sequencing allows plant biologists to generate increasingly contiguous assemblies (Jain, Olsen, Paten, & Akeson, 2016; Kersey, 2019). In forest trees, recent high‐quality assemblies include *Eucalyptus grandis* (Myburg et al., 2014), *Quercus robur* (Plomion et al., 2016, 2018), and *Liriodendron chinense* (Chen, Kao, Li, Huang, & Li, 2018). These inexpensive and beneficial sequencing methods are also attractive for the large and repetitive conifer genome assemblies that remain fragmented (Prunier, Verta, & MacKay, 2016). Reducing the challenges associated with sequencing and assembling a genome opens the door to the generation of pangenomes (Golicz, Batley, & Edwards, 2016). This approach extends the analysis beyond the scope of allelic variation within genes and toward structural variants across populations. Pangenomes are now available for maize (Hirsch et al., 2014), *Oryza* (Zhao et al., 2018), *Brassica* (Golicz et al., 2016), and *Brachypodium* (Gordon et al., 2017), and in trees, several hybridizing *Populus* species (Pinosio et al., 2016). While most research efforts are focused on diving deeper into species with economic drivers, fewer than 1% of the estimated 400,000 diverse land plants are sequenced. This may soon change as several initiatives are proposing ambitious collaborations to characterize large sections of the tree of life. The Earth BioGenome Project is most notable and intends to sequence 10 to 15 million eukaryotes over the next 10 years (Lewin et al., 2018). Obtaining high‐quality reference genomes for more forest tree species may improve our ability to conserve and manage forest populations.

Forest trees are long‐lived, predominantly outcrossing perennials with long generation times and tremendous genetic diversity. As such, a significant body of literature is dedicated to interrogating forest tree populations spanning environmental gradients through genomics (Aitken & Bemmels, 2016). Population studies examine local adaption through a range of techniques with recent efforts focused on reduced representation genome sampling (Catchen et al., 2017). To date, at least 50 tree species were assessed via genotyping‐by‐sequencing approaches, such as RAD‐Seq, which is a reliable option for trees with and without a reference genome (Parchman, Jahner, Uckele, Galland, & Eckert, 2018). These approaches are typically paired with extensive phenotypic or environmental data to interrogate genotype–phenotype and/or genotype–environment associations for a large number of individuals (Sork et al., 2013). The associated phenotypic and environmental metrics add yet another dimension to the data challenge. High‐throughput phenotyping, or phenomics, is extensively adopted in crop species to examine and monitor biomass, photosynthetic efficiency, disease status, growth traits, and root architecture (Fernandez, Bao, Tang, & Schnable, 2017; Shakoor, Lee, & Mockler, 2017; Thomas et al., 2016). Recent adoption of thermal imaging and LIDAR provides opportunities to assess biodiversity, response to drought, growth traits, and pest/pathogen spread across entire forest plots (Dungey et al., 2018; Ludovisi et al., 2017). This review will describe developments in cyberinfrastructure that enable integration across traditional domains to advance knowledge in the forest tree research community.

## 2 CYBERINFRASTRUCTURE AND FAIR

The term cyberinfrastructure was first defined by the National Science Foundation (NSF) in 2003, and described a research network that supported all aspects of the data life cycle, from acquisition to storage, integration, analysis, and visualization. Cyberinfrastructure includes both the software and hardware elements to support these endeavors, connected to the Internet and accessible to an audience beyond a single institution (Kim, Yu, & Park, 2016). There is agreement among many that these frameworks and the underlying data should be open, integrated, current, reproducible, and sustainable (Eiserhardt et al. 2018). The subsequent NSF investment in the iPlant Collaborative, now known as CyVerse, addresses challenges surrounding access to analytics, storage, and visualization for plant biologists (Goff et al., 2011; Stein, 2008). Rebranded in 2015 to serve the entire life science community, CyVerse provides computational infrastructure through cloud and virtual machines for common bioinformatic workflows as well as dedicated applications for data storage, training, and image analysis. The origins of CyVerse in plant biology provide a platform that remains accessible for those studying non‐model plants. Reproducible workflows are well integrated for de novo genome and transcriptome assemblies, variant detection, and targeted capture analysis (Horvath et al., 2018). While CyVerse focuses primarily on genomics, the BIEN (Botanical Information and Ecology Network), a National Center for Ecological Analysis and Synthesis (NCEAS) working group, unifies disparate ecological datasets built from observations across regional plots, herbaria, and other collections (Enquist, Condit, Peet, Schildhauer, & Thiers, 2016). The challenges are immense with varying degrees of digitization, distributed nonintegrated databases, and a lack of universally adopted standards for recording observations. BIEN is targeting not only integration across these collections but also phylogenetic and ‘omic data to fully assess the impact of climate change (Enquist et al., 2016). CyVerse and BIEN represent two coordinated efforts from which forest tree researchers could benefit. Integration of ecological, trait, and genomic data for georeferenced populations, alongside computational resources, is critical for questions surrounding forest health and productivity.

Cyberinfrastructure is only as powerful as the underlying data that it stores, transports, and analyzes. While this remains challenging for genetic and genomic data, it is even more so for field observations and measured traits. The FAIR (Findability, Accessibility, Interoperability, and Reusability) data reporting standards, published in 2016, emphasized that data should not only be stored, but also accessible and usable by the greater research community (Wilkinson et al., 2016). These guidelines encourage individual researchers seek out appropriate tools and cyberinfrastructure to support the viability of their digital products. The FAIR reporting standards ask that data be: (a) findable: requires that the information is both machine and human‐readable with relevant and persistent identifiers; (b) accessible: requires that information be indexed, searchable, and retrievable by both machines and humans through the use of open‐source standard file formats; (c) interoperable: requires that information be exchanged across platforms and relies on standards and semantics to aid in this process; (d) reusable: requires that data are open and associated with appropriate metadata (Reiser, Harper, Freeling, Han, & Luan, 2018). In the era of high‐throughput data, cyberinfrastructure and the associated databases are not yet fully compliant with FAIR standards.

## 3 WHERE ARE THE DATA?

Journals and funding agencies encourage the deposition of data in the appropriate archiving locations. Despite these guidelines, all life science fields are experiencing a decrease in well‐connected datasets (Alexander, Johnson, & Brown, 2018). In non‐model species, the derived genomic datasets are as important, if not more so, than the original raw reads. These derived datasets may include whole‐genome assemblies, intermediate assemblies from reduced representation population studies, *de novo* transcriptome assemblies, variant information, and genotyping assay designs. For example, of the 3,133 registered BioProject studies associated with *de novo* transcriptomics, only 374 projects representing 73 unique species are associated with a Transcriptome Shotgun Assembly (TSA) database record and/or Gene Expression Omnibus record in NCBI. This disconnect provides no mechanism to reproduce or reuse a transcriptome assembly. Even when datasets are connected, the associated metadata is typically sparse and does not include relevant details on the analytics involved in generating the connected datasets.

Outside of genomic data, the situation is far more dire; the vast majority of this content, if submitted, is associated with generalist repositories, such as Dryad (https://datadryad.org/), Zenodo (https://zenodo.org/), or FigShare (https://figshare.com/). These systems provide a long‐term Digital Object Identifier (DOI), but the hosted data are generally not discoverable or machine‐readable. These repositories accept data across a wide range of disciplines, in numerous formats, and provide little guidance or requirements on the submitter (Reiser et al., 2018). As an example, phenotypic and environmental data associated with forest tree populations can be found in Dryad for over 43 forest tree species. In all cases, the flat files referenced with the study are not provided in a machine‐readable format. Some of the best practices for trait data include designation of units for trait measures, detailed trait measure descriptions, definition of missing data, and *tidy* format (one row = one observation, one column = one trait) (Wilson et al., 2017).

For plant biologists, the ELIXIR UK‐supported Collaborative Open Plant Omics (COPO) initiative is improving the situation for plant genomic and phenomic data with standards‐based integration, guided workflows, DOI generation, and connections to researcher profiles (ORCID) (Shaw et al., 2015). The COPO initiative aims to limit the variation across standards and provide access to analytics which can operate on more robust standards. COPO is just one of the registered services listed in the Fairsharing.org portal that provides a curated and queryable interface to four linked registries, including data standards, databases, collections, and data policies (McQuilton et al., 2016). FAIRSharing is aligned with the FAIR principles and provides guidance on data sharing for numerous disciplines, including the life sciences, for individual researchers, journals, and funding agencies.

## 4 DATABASES

Primary databases are long‐term, federally funded, entities that are capable of maintaining persistent identifiers. The major representatives in the genetic and genomic world include NCBI GenBank, EMBL‐EBI, and DDBJ, which operate as mirrored repositories for several different sequence types with independent strengths (Meldal & Orchard, 2018; Miyazawa, 2018; Sayers et al., 2018). These repositories excel at providing unified access to a wide range of sequence data for an unlimited number of species. They do not, however, have the capacity to identify specific community needs and provide organism‐specific curation. They generally provide basic functionality for sequence search, sequence comparison (BLAST), and visualization (genome browsers). Since users are often seeking substantial volumes of data, they provide mechanisms for bulk download via FTP, command‐line search, and Web‐based searches, as well as rapid data transfer pipelines, such as Aspera. In addition, primary repositories must balance data volumes and perceived benefit to the research community. They implement policies that are typically driven by biomedical and model system concerns. This includes NCBI’s recent decision to halt the collection of variant data from genomes that are not biomedical models and within EBI‐EMBL, only variants associated with INSDC‐registered genomes. For those species with draft genomes or without a reference, this provides no mechanism for integrated data sharing of population genomic studies.

Secondary databases curate and provide specialized functionality to clade, family, or species‐specific research communities. They operate in conjunction with primary databases and have a long history of cross‐linking resources. They are frequently funded by organizations with a data production goal from a multi‐institutional project with a dedicated team of biocurators. In the broader plant genomic arena, the Joint Genome Institute's Phytozome resource hosts versioned genomes via JBrowse across the viridiplantae as well as structural and functional annotation downloads (Buels et al., 2016; Goodstein et al., 2011). The complementary but independent PLAZA plant comparative genomic framework focuses on different plant clades with instances for monocot, dicot, and gymnosperm datasets. Each instance contains curated structural and functional gene annotations, gene family comparisons, and phylogenetic analysis (Van Bel et al., 2017). Other comparative genomic resources such as Gramene and Ensembl Plants provide extensive resources for comparative genomics, pathway analysis, and variant data, but rely on high‐quality reference genomes from crop species and house few tree species (Bolser, Staines, Pritchard, & Kersey, 2016; Tello‐Ruiz et al., 2017). Large‐scale collaborative projects are also generating and hosting substantial Web‐based genomic resources. The international 1KP project generated de novo reference transcriptomes for over 1,000 viridiplantae species with membership from all major lineages (Matasci et al., 2014). The successor to this project, in collaboration with Earth BioGenome, is the Plant 10KB project, which will sequence 10,000 phylogenetically diverse plant species from major clades of embryophytes over the next five years (Cheng et al., 2018). Independently, these resources provide valuable contributions to forest tree genomic research; however, connections between these resources and non‐model databases remain sparse.

Community databases also work in conjunction with primary databases and other secondary databases. Their origins are more ad hoc in that they are hosted by a variety of different organizations and funded through different mechanisms. The Arabidopsis Information Resource (TAIR) is a well‐established community repository for plant biologists that provides a wealth of information that is also cross‐linked across information resources (Berardini et al., 2015). Community and secondary databases for plants (and trees) continue to increase and often originate from a single transcriptome or genome project; however, dedicated funding for biocuration beyond the length of the initial project is limited (Harper et al., 2018). Until recently, the majority of databases focused on interfaces for searching curated data, genome visualization via browser, and basic sequence similarity functions. The tree biologist's need for cyberinfrastructure that expands the basic search and BLAST functionality of community databases is responsible for recent and successful deployments of more robust frameworks.

Three Web‐based forest tree repositories have persisted with independent specialties and a connection to data analytics: TreeGenes, Hardwood Genomics Web, and three PlantGenIE implementations (Table 1). Both TreeGenes and Hardwood Genomics Web serve as hubs for their respective research communities in addition to the role of data storage, access, and analysis (Chen et al., 2017; Falk et al., 2018). Combined, they host over 1,800 species with the goal of providing integrated resources for non‐model forest trees. Hardwood Genomics Web provides expression and co‐expression analysis support for model and non‐model hardwood species (Chen et al., 2017). TreeGenes supports population and landscape genomic analysis as well as comparative genomic module for orthologous gene family analysis. Recent development in both is focused on the Tripal framework. This open‐source platform combines a content management system front end with an organism agnostic relational database schema, known as Chado (Sanderson et al., 2013; Spoor et al. 2019). This web/database combination provides a set of modules that can load and provide public views for standard data types (genomes, transcripts, variants, etc). Two other prominent tree databases associated with horticultural species, CitrusDB and Genome Database for Rosaceae (GDR), utilize Tripal as well as 30 other plant‐focused resources (Jung et al., 2018, 2017). Tripal‐supported databases integrate with a community of developers that contribute modules to extend the functionality of the standard install (Zhou, Emmert, & Zhang, 2005). In the forest tree Tripal instances, genetic maps are served through the Comparative Genetic Map module, genomes through the JBrowse module, and variants in the Natural Diversity module (Jung et al., 2011; Youens‐Clark, Faga, Yap, Stein, & Ware, 2009). Custom Tripal modules, such as CartograTree in TreeGenes, provide analytics for association genetics and landscape genomic studies through the large‐scale integration of genetic, phenotypic and environmental data (Falk et al., 2018).

**Table 1:** Database resources for tree species

Database	Start date	Scope	Data sharing	URL	Ontologies	FAIR sharing	Analytics
TreeGenes	1995	Forest Trees	Tripal/Elastic Search	http://treegenesdb.org	SO, GO, TO, PO, CO, PATO, CHEBI	X	X
Gramene	2001	Plantae	BioMart/Expression Atlas	http://www.gramene.org	SO, GO, PO, EFO	X	X
Genome Database for Rosaceae	2004	*Rosaceae*	Tripal/Elastic Search	https://www.rosaceae.org	SO, GO, TO, PO, CO, PATO	X	X
TropGeneDB	2004	Tropical Trees	N/A	http://tropgenedb.cirad.fr	SO, GO	X	
AspenDB	2004	*Populus*	N/A	http://aspendb.uga.edu	SO, GO		X
PopGenIE	2009	*Populus*	PlantGenIE	http://popgenie.org	SO, GO		X
PLAZA	2009	Plantae	N/A	https://bioinformatics.psb.ugent.be/plaza	SO, GO	X	X
Rubber Tree Genome	2010	*Hevea*	N/A	http://www4a.biotec.or.th/rubber	SO, GO		
Citrus Genome Database	2011	*Citrus*	Tripal/Elastic Search	https://www.citrusgenomedb.org	SO, GO, TO, PO, CO, PATO	X	X
CsiDB	2011	*Citrus*	N/A	http://citrus.hzau.edu.cn/orange	SO, GO		X
EucGenIE	2011	*Eucalyptus*	PlantGenIE	https://eucgenie.org	SO, GO		X
Eucalyptus camaldulensis Genome Database	2011	*Eucalyptus*	N/A	http://www.kazusa.or.jp/eucaly	SO, GO		
Jatropha Genome Database	2011	*Jatropha*	N/A	https://www.kazusa.or.jp/jatropha	SO, GO		
Phytozome	2012	Viridiplantae	InterMine	https://phytozome.jgi.doe.gov/pz/portal.html	SO, GO	X	
ConGenIE	2013	Conifers	PlantGenIE	http://congenie.org	SO, GO		X
Pear Genome Project	2013	*Pyrus*	N/A	peargenome.njau.edu.cn	SO, GO		
TropiTree	2014	Tropical Trees	N/A	https://ics.hutton.ac.uk/tropiTree/index.html	SO, GO		
PGDBj	2014	Plantae	N/A	http://pgdbj.jp	SO, GO	X	X
Hardwood Genomics Web	2015	Hardwood Forest Trees	Tripal/Elastic Search	https://www.hardwoodgenomics.org	SO, GO, TO, PO, CO, PATO	X	X
PGP Repository	2015	Plantae	e!DAL	http://edal-pgp.ipk-gatersleben.de/		X	
Quercus Portal	2015	*Quercus*	N/A	https://quercusportal.pierroton.inra.fr	SO, GO		
Ash Tree Genomes	2016	*Fraxinus*	N/A	http://www.ashgenome.org	SO, GO		
GDA: genome database for angiosperms	2016	Angiosperms	N/A	http://www.angiosperms.org	SO, GO		
Jatropha Curcas Database	2016	*Jatropha*	N/A	http://jcdb.xtbg.ac.cn/	SO, GO		
Valley Oak Genome Project	2016	*Quercus*	N/A	https://valleyoak.ucla.edu	SO, GO		
EUCANEXT	2017	*Eucalyptus*	N/A	http://bioinfo03.ibi.unicamp.br/eucalyptusdb	SO, GO		X
Rubber Genome and Transcriptome DB	2017	*Hevea*	N/A	http://matsui-lab.riken.jp/rubber/home.html	SO, GO		X
Citrus Greening Database	2017	*Citrus*	N/A	http://citrusgreening.org	SO, GO	X	X
Cacao Genome Database	2018	*Theobroma*	Tripal	https://www.cacaogenomedb.org	SO, GO, TO, PO, CO, PATO	X	X
CorkOakDB	2018	*Quercus*	Tripal	http://corkoakdb.org	SO, GO		X

The Plant Genome Integrative Explorer (PlantGenIE) framework supports three dedicated forest tree genus/family‐specific domains: *Populus* (PopGenIE), Conifer (ConGenIE), and *Eucalyptus *(EucGenIE; Sundell et al., 2015). Each domain is packaged with a set of core tools that are a combination of GMOD project tools, such as JBrowse and Apollo, and standard open‐source tools, such as BLAST, and custom tools. The custom tools are focused on the large‐scale expression study visualization, analysis, such as GO enrichment, and co‐expression evaluation (Sundell et al., 2015). Current development is focused on the integration of ChiP‐Seq, variant, and small RNA (sRNA) datasets. Both the Tripal and PlantGenIE platforms provide cross‐site search capabilities and a core framework that allows rapid development of a new instance. Tripal implements cross‐site search with an Elastic Search module that removes dependence on a uniform database schema (Condon, Almsaeed, Chen, West, & Staton, 2018). For example, all of the Tripal tree databases are currently running the Elastic Search module, which allows a visitor to TreeGenes to search a specific gene and request that matching results are returned from Hardwood Genomics Web, CitrusDB, and Genome Database for Rosaceae. PlantGenIE databases accomplish cross‐site query through a shared underlying schema. Both frameworks support analytic capacity through the open‐source Galaxy framework (Boekel et al., 2015). Galaxy exists as a publicly accessible Web framework with community‐curated workflows for a wide range of bioinformatic analysis. It is also an international consortium of developers that support local instances that can be further customized for a variety of community needs. Community databases can manage user accounts, provide data storage, and expose custom workflows and associated datasets through their sites with Galaxy.

For model organisms, data warehousing solutions can enable faster access through alternative (non‐relational) storage designs. BioMART is widely adopted and provides an efficient storage method and standardized user interface to query genomic objects, including genes and functional data (Smedley et al., 2015). BioMART also pairs with an R package that allows one to integrate functional annotations directly into analytics (Drost & Paszkowski, 2017). Gramene, Phytozome, and Ensembl Plants provide data access via BioMART in addition to their independent interfaces. InterMine acts as a more robust framework that combines efficient storage with standard and custom data loaders and analytic tools (Lyne et al., 2015). The Phytozome framework also implements a PhytoMine. As demonstrated by Phytozome, databases have the option to share or expose data in different frameworks, which can enable a variety of Application Programming Interfaces (APIs), other databases, or end users to integrate the data.

**Figure 3:**
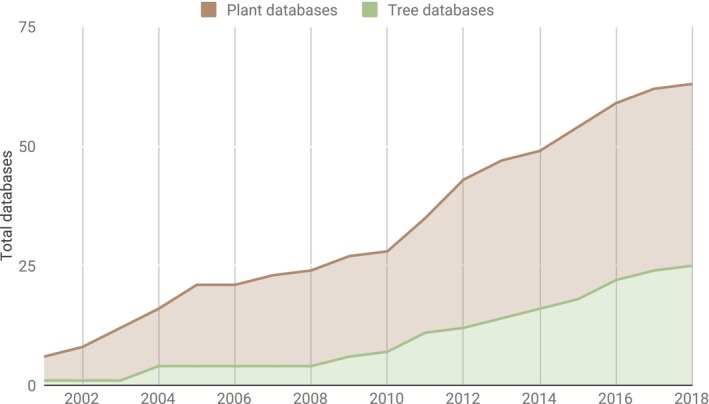
Plant and tree‐specific secondary and community databases from 2002 to present

In alignment with FAIR guidelines, new tree (or plant) community databases should examine whether an independent Web resource is necessary or whether integration into existing cyberinfrastructure is more sustainable. The support of advanced analytics in community databases encourages frameworks to efficiently transfer data, such as raw reads, from primary repositories to local application servers for analysis. In the era of big data, it is not efficient or realistic to reinvent the functionality required for each new genome or transcriptome. If a new and independent database is required, researchers should consider how to share the data during the lifetime of the resource as well as a plan to disseminate the data in the event it can no longer be maintained as an independent resource. Less than half of the existing databases hosting tree related data are considering aspects of FAIR, and just over half are providing access to basic analytics (Table 1, Figure 3). In the biomedical community, a pilot NIH Data Commons initiative is seeking to integrate independent genomic resources, including Flybase, Mouse Genome Database (MGD), Wormbase, and others into a cloud‐based data sharing platform to minimize redundancy and improve integration and data reuse (Mahurkar et al., 2018). Related to species of agricultural interest, the AgBioData consortium, formed in 2015, represents more than 25 genetic, genomic, and breeding databases hosted in a range of platforms. The consortium values the need for biocuration and encourages member databases to think about data sharing, reuse, and sustainability for existing resources (Harper et al., 2018). Community databases should coordinate with existing consortiums to improve visibility, reduce development time, contribute to open source projects, and extend the value of their resource. For projects consisting of a single genome reference and associated transcriptome, project members should explore the options for working with an existing resource.

## 5 ONTOLOGIES AND STANDARDS

Datasets curated by biologists have concepts and measures associated with different definitions across disciplines, organisms, scales, and even researchers in the same field. This semantic heterogeneity impedes data integration. Standardized vocabularies, known as ontologies, are used to describe genetic, phenotypic, and environmental observations or products (Bard & Rhee, 2004). The Gene Ontology (GO) Consortium is a well‐established effort in the life sciences that provides curated, relational‐term databases describing the functions, processes, and cellular locations of gene products (The Gene Ontology Consortium, 2019). The standard acyclic graph organization of ontologies, paired with persistent identifiers for terms, supports connections within and between databases. GO and the Sequence Ontology (SO), which defines the meaning of words such as gene, linkage group, and variant, are adopted by the majority of databases and frameworks, including InterMine and BioMART (Cunningham, Moore, Ruiz‐Schultz, Ritchie, & Eilbeck, 2015; Eilbeck et al., 2005). Today, one can visit the OBO Foundry and EMBL‐EBI’s Ontology Lookup Service to search the curated bio‐ontologies, of which over 30 are specific to plants (Vita, Overton, Mungall, Sette, & Peters, 2018). Among these, five, in addition to the Crop Ontology (CO), are extensively used by databases supporting plant research (Table 2). These ontologies are structured and semantically formalized to promote integration into data standard recommendations. The Plant Trait Ontology (TO), dedicated to phenotypes, the Plant Ontology (PO), dedicated to structures and developmental stages, and the CO for breeding, germplasm, and traits organized by species are most active (Arnaud et al., 2012; Cooper et al., 2013; Shrestha et al., 2012). The ecology and phylogenetic community recently released the Plant Phenology Ontology (PPO) and Flora Phenotype Ontology (FLOPO). The FLOPO is focused on morphological structures and traits associated with their identification, value, and application (Hoehndorf et al., 2016). The PPO will address challenges associated with the varied vocabularies applied to digitized collections and phenological measures across scales (Stucky et al., 2018). The PPO is intended to serve three existing networks: USA National Phenology Network (USA‐NPN), National Ecological Observatory Network (NEON), and the Pan‐European Phenology Database (PEP725). A related effort, described as a Thesaurus of Plant Characteristics (TOP), is intended for use in the Plant Trait Database (TRY‐DB), which is a global archive for curated plant traits (Garnier et al., 2017). The TRY‐DB is uniquely positioned to improve on the data integration challenges associated with ecological trait data by hosting over 6.9 million trait records for 148,000 plant taxa (Kattge et al., 2011).

**Table 2:** Reference ontologies/vocabularies for plants

Ontology Name	Scope	Unique Terms
Crop Ontology (CO)^a^	Traits (species‐specific)	6298
Trait Ontology (TO)^a^	Trait	1554
Plant Ontology (PO)^a^	Anatomy and development	1991
Gene Ontology (GO)^a^	Gene product	49993
Phenotypic Qualities Ontology (PATO)^a^	Trait qualities	2730
Chemical Entities of Biological Interest (CHEBI)	Chemistry	132780
Plant Experimental Conditions Ontology (PECO)	Environment	563
Sequence Ontology (SO)^a^	Genetic	2473
Protein Ontology (PRO)	Protein products	216442
Plant Phenology Ontology (PPO)	Phenology	254
Flora Phenotype Ontology (FLOPO)	Morphology and trait	24199

a Ontologies widely adopted in plant genetic databases.

The Planteome initiative provides a Web portal with interconnected reference ontologies for the annotation of genomes, expression data, germplasm, and traits for 95 taxa (Cooper et al., 2018). The reference ontologies include PO, TO, and Plant Experimental Conditions Ontology (PECO), and the in‐development Plant Stress Ontology (PSO), which will describe the abiotic and biotic stressors. These are integrated with additional terms from CO, GO, Chemical Entities of Biological Interest (ChEBI), Evidence and Conclusion Ontology (PECO), and the Phenotypic Qualities Ontology (PATO) (Cooper et al., 2018). PATO unifies phenotype descriptions and makes them amenable to automated processing. It is both an ontology and a uniform way to express phenotype statements (Gkoutos, Schofield, & Hoehndorf, 2017). Planteome leverages the integrated platform to provide annotations which connect an ontology term to a bioentity. A bioentity is defined as a QTL, gene, protein, germplasm, gene product, or similar.

Two independent efforts have brought tree biologists and computational teams to the same table to curate traits and structures. A wood anatomy and development working group contributed to PO through the partial conversion of established vocabularies in the Glossary of Terms used in Wood Anatomy (Lens et al., 2012). While this glossary is known to the research community, the term definitions lose meaning when adopted in other disciplines classifying the same structures. Within the CO, an INRA‐sponsored effort curated a woody plant trait ontology specific to forest tree breeding and health with terms such as wood density, wood fiber length, tree diameter, and branching angles. This ontology provides a much‐needed standard for structures and traits specific to forest trees that are not represented in other crop species.

The combination of plant‐specific and reference ontologies utilized in databases leverages curated efforts and minimizes redundancy. The transition of community‐specific vocabularies or natural language descriptions into ontology terms enables automation of aspects of the classification process. While biocuration is an important and fundamental activity for all life science databases, there are few reliable funding streams that will support it (Harper et al., 2018). Community databases must select the ontologies that are appropriate for the data they hold and consider workflows to assist in automating data annotation from high‐throughput studies. The availability of terms appropriate for woody plant species vastly improves the ability to annotate population and expression studies available to forest tree databases. Reference ontologies applied to the sequence objects, gene products, plant traits/phenotypes, and environmental metrics describe a complete study in a manner that should be easily indexed, searched, integrated, and compared.

The FAIRSharing portal provides details on the registered databases and the ontologies they currently support. Ontologies play an important role in the implementation of FAIR guidelines as they define shared vocabularies and improve the machine‐readable aspect of data. Structured standards, such as the Minimal Information About a Plant Phenotyping Experiment (MIAPPE), provide guidelines for reporting on a phenotyping experiment (Ćwiek‐Kupczyńska et al., 2016). These guidelines integrate across all of the active plant ontologies and several of the reference ontologies named here. The MIAPPE standards are flexible to landscape, greenhouse, growth chamber, and related study designs. They formalize the language around the design, treatments, environmental metrics, plant structures, and traits. Ontologies, integrated with standards and connected to guided workflows, as proposed by COPO, are critical for reusable data in community databases. Recent efforts, focused on high‐throughout phenotyping, include GnpIS, which serves as a FAIR, international information resource for integrating phenomic, genomic, and metadata for plants and plant pathogens (Pommier et al., 2019). Their efforts have extended outside of species with well‐resolved genomes and include several forest trees. Coordinated development with workflows enforcing FAIR standards and community databases is still needed. Individual researchers do not have the time and resources to map terms onto complex ontological frameworks. In addition, each ontology requires independent teams to generate term annotations and curate updates. Once the ontology is established, intelligent workflows for data submission can ease the burden on researchers and curation teams, while increasing the value of the data itself. Independent and consistent support of the ontologies, the data submission frameworks, and the community databases is needed to ensure robust integration. Fields such as ecological genomics are relevant for forest trees and are driving the need for reporting standards that can integrate across scales (Farley, Dawson, Goring, & Williams, 2018). Effective integration of metadata standards, data sharing implementations, and ontological frameworks provides the basis for tools such as CartograTree that enable meta‐analysis across population studies for forest trees (Falk et al., 2018).

## 6 WORKFLOWS AND ANALYTICS

The strength of the bioinformatic research community is the active open‐source development that provides innovative solutions to address analytic challenges. On the downside, this creates an environment where the software versions change rapidly, possibly several times a year, and the best package for the task may change just as often. For the average tree biologist focused on fieldwork, sampling, sequencing, and analysis, the nuances of the latest and greatest approach are difficult and overwhelming to track. This environment, combined with software that is typically developed for Linux systems on HPC, provides additional hurdles for the end user. Access to computing resources in research institutions has improved, but hurdles in installing and maintaining packages that require frequent updates and navigating complex scheduler software to submit jobs remain a challenge. Cyberinfrastructure seeks to connect data and analytics, which can be facilitated through vetted workflows. Workflow languages provide a variety of options, with command‐line and Web‐based implementations, as well as the ability to wrap existing packages (Table 3). For community databases and end users, workflows implemented in workbenches, such as Galaxy, Taverna, or SciApps, offer the ability for end users with less development experience to work within a graphical user interface to design modular workflows that wrap existing open‐source bioinformatic tools (Boekel et al., 2015; Leipzig, 2017; Wang, Lu, Buren, & Ware, 2018; Wolstencroft et al., 2013). Community databases can integrate with local (or public) instances of these workbenches to expose workflows to their user community. The Tripal community provides this resource to all member databases and their users via Galaxy. Customization of the workflows in tools such as Galaxy allows database administrators to expose and update best practice workflows as well as provide HPC access. This is of tremendous importance for non‐model plant databases where custom workflows that do not rely on reference genomes must communicate with curated, local genomic resources. For both models and non‐models, integration of genomic selection workflows with management tools for breeding could produce robust infrastructure for the full life cycle in forestry. Following the execution of a workflow hosted by a community database, tools such as CyVerse's Data Store can provide indexed and labeled storage with support for authentication, permissions management, and metadata associations (Schneider & Jimenez, 2019).

**Table 3:** Workflow languages to support bioinformatic analysis

Workflow Language/Workbench	Year	Web‐based/Command Line	Syntax
Apache Taverna	2004	Both	Explicit
Pegasus	2005	Command Line	Explicit
Ruffus	2010	Command Line	Explicit
Galaxy	2010	Both	Explicit
Snakemake	2012	Command Line	Implicit
bpipe	2012	Command Line	Explicit
Agave	2012	Both	Explicit
BigDataScript	2015	Command Line	Implicit
Sci:Luigi	2016	Command Line	Implicit
Common Workflow Language	2016	Both	Explicit
Nextflow	2017	Both	Implicit
Toil	2017	Command Line	Explicit

## 7 CYBERINFRASTRUCTURE RECOMMENDATIONS FOR THE FUTURE

**Figure 4:**
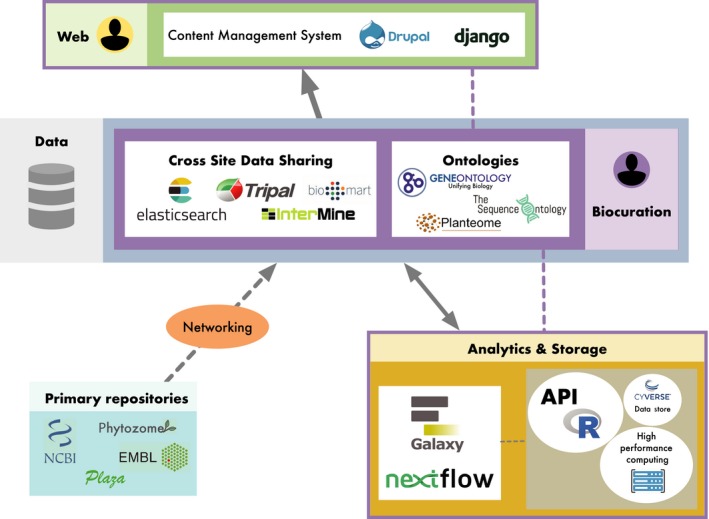
Schematic of recommended cyberinfrastructure to support and integrate non‐model tree genomics, phenomics, and environmental data. Community databases housed within existing frameworks that utilize content management systems will ease the management of user accounts, data exchange, and content updates. Guided submission workflows will integrate community‐curated ontologies, such as GO, SO, PO, TO, CO, and PATO. Regular imports from primary and secondary sources, as well as multi‐institutional projects, will provide the basis for data that can be further curated. Registered users will have direct access to custom workflows with data housed in the database and raw data that can be transferred from primary databases to the local application server

The era of high‐throughput data necessitates efforts to minimize redundancy in storage and optimize methods for finding and reusing the information generated (Figure 4). While genomic data are better positioned for integration among model systems, the current state for non‐models is less than ideal. With the upcoming increase in draft and complete genome references for species without a substantial research community, integration of these resources into established frameworks, such as Tripal, PlantGenIE, InterMine, or BioMART, is important in an era of limited public funds for computational resources. Community databases should consider exposing data in more than one semantic framework to maximize data sharing, and new databases should develop their resource in one of the community‐supported open‐source frameworks to minimize developer time and leverage established standards. Integration of ontological frameworks with guided submission workflows that can capture and label metadata (study design, geographic data, and analytical methods) is key to generating reusable and reproducible datasets. These guided workflows can enforce submission of both the raw data and derived objects to ensure they are well described and accessible. Generation of persistent identifiers (DOIs) will also be required to provide lasting value to the associated digital objects. They should be designed to provide at least partial automation for term assignments for sequence types, gene products, and phenotypes.

We expect that many of our forest tree species will have a reference genome in the next five to ten years. As such, the ability to integrate decades of population studies onto these genomes will be critical. In addition, we will want to leverage efforts, such as Planteome, to aid in the functional annotation of the gene space. Journals and funding agencies, in collaboration with initiatives such as FAIRSharing, must continue their role as gatekeepers and determine best practices and preferred standards for specific data types. Agreement on best practices and enforcement of these standards for both publications and data management plans remains a significant barrier. Researchers and funding agencies should look to existing cyberinfrastructure solutions to manage projects from start to finish, rather than at the end of the project. Metadata tagging, ontology term assignment, and raw data storage can be managed during small‐ or large‐scale collaborations. Many community databases and other information resources can support this activity and keep data accessible only to project members until public release (Pommier et al., 2019; Wegrzyn et al., 2019).

Finally, reproducible, documented, and custom analytic workflows should be accessible to researchers through the community databases that provide the curated datasets. These integrated platforms must be accessible in the field as a data and metadata collection tool and at the desktop to provide analysis, visualization, and submission. Mobile applications for data collection on the landscape as well as in tree plantations are a key element of cyberinfrastructure (Crocker et al., 2019). Machine learning supported workflows to distill information from high‐throughput phenotyping via remote sensing, an increasingly important component of data collection for forest health and productivity, will be required (Kälin, Lang, Hug, Gessler, & Wegner, 2019). Forest tree research will benefit from well‐connected and labeled datasets with access to analytics that can integrate across genomic, phenomic, and environmental data.

## CONFLICT OF INTEREST

None declared.

## Data Availability

The data that support the findings of this study are openly available in NCBI, FAIRSharing, Data Dryad at https://www.ncbi.nlm.nih.gov/genbank/, http://FAIRSharing.org, and https://datadryad.org/
